# Genome-wide association study to identify novel loci and genes for Fusarium root rot resistance in sweet potato using genotyping-by-sequencing

**DOI:** 10.3389/fpls.2023.1251157

**Published:** 2023-10-04

**Authors:** Tae Hwa Kim, Sujung Kim, Won Park, Koan Sik Woo, Keunpyo Lee, Mi Nam Chung, Young Hoon Lee, Hyeong-Un Lee, Kyo Hwui Lee, Sang-Sik Nam, Hyun Jo, Jeong-Dong Lee

**Affiliations:** ^1^Bioenergy Crop Research Institute, National Institute of Crop Science, Rural Development Administration, Muan, Republic of Korea; ^2^International Technology Cooperation Center, Technology Cooperation Bureau, Rural Development Administration, Jeonju, Republic of Korea; ^3^Planning and Coordination Division, National Institute of Crop Science, Rural Development Administration, Jeonju, Republic of Korea; ^4^Department of Applied Biosciences, Kyungpook National University, Daegu, Republic of Korea

**Keywords:** sweet potato, Fusarium root rot, resistance, genome-wide association study, genotyping-by-sequencing

## Abstract

Fusarium root rot, caused by *Fusarium solani*, is a major post-harvest disease in sweet potatoes (*Ipomoea batatas* (L.) Lam.). An effective strategy for controlling this disease is the development of resistant varieties. In this study, a genome-wide association study (GWAS) was conducted on 96 sweet potato genotypes to identify novel candidate loci and dissect the genetic basis of Fusarium root rot resistance. Genotyping was performed using genotyping-by-sequencing (GBS), and 44,255 SNPs were identified after filtering. The genotypes (n = 96) were evaluated through resistance tests in 2021 and 2022, separately and combined. The GWAS identified two significant SNP markers (LG3_22903756 and LG4_2449919) on chromosomes 3 and 4 associated with Fusarium root rot resistance, respectively. Lesion length showed significant differences between homozygous A and G alleles of LG3_22903756, which can potentially be used to develop molecular markers for selecting accessions resistant to Fusarium root rot. Expression analysis of 11 putative genes flanking the significant SNPs revealed the alteration in the expression of nine genes, indicating their possible involvement in Fusarium root rot resistance. The results of this study will aid in the marker-assisted selection and functional analysis of candidate genes for Fusarium root rot resistance in sweet potatoes.

## 1 Introduction

Sweet potato (*Ipomoea batatas* (L.) Lam.) is an essential crop cultivated in more than 7.4 million ha, with more than 88 million tons of annual production in 2021 (FAOSTAT, 2023). Sweet potato has long been considered an energy crop and is a healthy food because of its abundant nutrients such as carbohydrates, protein, fiber, and vitamins, especially anthocyanin and carotenoids (Alam, 2021). Sweet potatoes must be stored long after harvest to increase their sweetness through starch degradation. However, post-harvest losses during storage are caused by physical damage, weight loss, sprouting, and diseases (Tomlins et al., 2002; Ray and Ravi, 2005). Loss of storage roots during storage periods is primarily because of infection by soil microorganisms, and the disease worsens owing to careless handling.

Fusarium root rot is a serious post-harvest disease caused by *Fusarium solani* present in the soil of storage roots (Kim et al., 2022). Storage roots are wounded during harvesting or processing, and pathogens invade these wounds (Scruggs and Quesada-Ocampo, 2016). Fusarium root rot lesions are circular with dark brown concentric rings and may extend into the epidermis. White mycelia are formed by dried hollows inside the roots (da Silva and Clark, 2013). Pathogens from diseased roots are transmitted to the near-storage roots through contact with tools, hands, or water, increasing the damage. *Fusarium solani* is a soil-borne pathogen that has a wide range of hosts, such as soybean, potato, tomato, and sweet potato (Clark, 1980; Lawrence et al., 1989; Ajilogba and Babalola, 2013; Bojanowski et al., 2013). In addition, the pathogen can grow at a wide range of temperatures (8.5–34.3 °C) and cause wilt and rot of leaves, stems, and roots during cultivation or storage, decreasing the production yield and quality of crops (Wang et al., 2014; Yan and Nelson, 2020; Bilgili et al., 2023).

Developing resistant cultivars is an effective and economical way to reduce losses caused by disease (Lee et al., 2019). Conventional breeding of sweet potatoes for a single trait, especially storage disease resistance, is complicated and time-consuming. Marker-assisted selection (MAS) is a method of selecting and developing cultivars with traits of interest using molecular markers by identification of quantitative trait loci (QTL) (He et al., 2014). In addition, MAS can reduce the time and effort required to eliminate lines lacking disease-resistance genes in early generations (Ragimekula et al., 2013). Genetic analysis must be performed to find information on major genes and QTL and develop the selection markers in marker-assisted breeding programs (Hasan et al., 2021). A study to identify QTL for Fusarium root rot in common beans has been conducted by the Andean and Middle American diversity panel, and potential candidate genes such as ethylene response factor 1 (*ERF1*) or *CYP450* genes were found (Zitnick-Anderson et al., 2020). Furthermore, QTL analysis for Fusarium head blight found *QFhb-2DL* (a KASP marker) and candidate genes such as ethylene-responsive transcription factor, disease resistance proteins RPM1 and cysteine, and histidine-rich-domain-containing protein (Li et al., 2022).

However, genetic studies on sweet potatoes have been challenging because of the complexity of its genome. Sweet potato, a hexaploid species with 90 chromosomes (2n = 6x = 90), is highly heterozygous, and its genome size is estimated to be approximately 4.8–5.3 pg/2C nucleus (Ozias-Akins and Jarret, 1994; Isobe et al., 2017). Sweet potatoes are sometimes cross-incompatible but generally self-incompatible, which limits the construction of genetic populations in this crop (Yan et al., 2022; Xiao et al., 2023). Furthermore, diverse genotypes are observed owing to a large number of combinations in F_1_ progenies, imposing challenges to genetic mapping and QTL analysis (Yan et al., 2022; Xiao et al., 2023). Studies on Fusarium root rot resistance loci are limited, and further research to clarify the Fusarium root rot resistance mechanism in sweet potatoes is required. Although only one QTL mapping study was conducted to identify the QTL for Fusarium root rot resistance with simple sequence repeat (SSR) markers for F_1_ populations, a few QTL were identified but remained unverified because of the lack of related data and the small number of markers (n = 300) (Ma et al., 2020). Recently, transcriptome analysis for sweet potato roots infected by *F. solani* showed that the number of upregulated genes was lower than that of downregulated genes such as calcium-binding protein, and many homologous genes, including 43 WRKY genes and 57 genes from the MYB family of proteins, were differently or inconsistently expressed, suggesting that genetic studies in sweet potatoes are challenging (Zhang et al., 2023).

A genome-wide association study (GWAS) is used to evaluate the association between genotypes and phenotypes of interest among individuals. GWAS does not require developing genetic populations; therefore, it is less time-consuming, and the results may not be limited to genetic populations (Korte and Farlow, 2013). In sweet potatoes, GWAS have been conducted on yield, weevil resistance, nematode resistance, and anthocyanin content using the genome sequence of diploid *I*. *trifida*, a close ancestor of the hexaploid sweet potato, owing to the absence of a reference genome sequence (Hirakawa et al., 2015; Okada et al., 2019; Haque et al., 2020; Obata et al., 2022). In addition, a genome-wide analysis of expression quantitative trait locus (eQTL) has been conducted for agronomic traits such as flavonoid biosynthesis and storage root flesh color with 86 accessions (Zhang et al., 2020). Moreover, a genome-wide association study for the sugar and starch contents of 66 germplasms has been performed, and seven sugar and starch metabolism-related genes were identified (Nie et al., 2023). In 2017, the haplotype-resolved sweet potato genome was sequenced and assembled with the cultivar Taizhong 6 and used for a GWAS on weevil resistance and root flesh color (Yang et al., 2017; Liu et al., 2022a; Liu et al., 2022b).

This study aimed to (1) evaluate the phenotypic responses to Fusarium root rot using 96 genotypes, (2) identify linked SNPs and candidate genes based on GWAS, and (3) analyze the expression of potential candidate genes for Fusarium root rot resistance in sweet potatoes. To the best of our knowledge, this is the first study using GWAS to identify the genomic regions and genes associated with Fusarium root rot in sweet potatoes.

## 2 Materials and methods

### 2.1 Pathogen isolation, identification, pathogenicity test

Diseased sweet potato storage roots were collected from storehouses in the main cultivation regions (Haenam, Muan, and Yeong-am, Korea) in 2021. Pathogens were isolated from the collected samples using a modified method described by Yang et al. (2018). The border between diseased and healthy tissue was cut into 5 × 5 mm and sterilized in 70% ethanol for 30 s, followed by 1% sodium hypochlorite for 30 s. Then, the samples were placed on the water agar medium and cultured at 25 °C for 5 d after washing three times with sterilized distilled water (SDW). The single spores were isolated from the mycelium, transferred onto potato dextrose agar (PDA) slant medium, and the isolates were stored at 4 °C.

To identify species of isolates, genomic DNA was extracted from the mycelium incubated on PDA at 25 °C for 7 d using the Genomic DNA Prep Kit for fungi (Solgent, Korea) (Paul et al., 2007). The polymerase chain reaction (PCR) amplification was conducted with internal transcribed spacer (ITS) 1 (5′-TCC GTA GGT GAA CCT GCG G-3′) and ITS4 (5′-TCC TCC GCT TAT TGA TAT GC-3′) using a previously reported method (White et al., 1990) under the following conditions: pre-denaturation at 94 °C for 1 min, followed by 30 cycles of denaturation at 94 °C for 30 s, primer annealing at 56 °C for 30 s, and extension at 72 °C for 1 min 40 s with a final elongation at 72 °C for 10 min. The PCR products were sequenced in both directions and used for phylogenic analysis and multiple sequencing alignment using ClustalW (Thompson et al., 1994) and BioEdit (v.7.2.5, Hall, 1999). Phylogenetic analysis was assessed using MEGA X (Saitou and Nei, 1987), and a neighbor-joining (NJ) phylogenetic tree was constructed for ITS rDNA sequences (Saitou and Nei, 1987; Kumar et al., 2018). Sequence identity was analyzed for each isolate using the Basic Local Alignment Search Tool (BLAST).

A pathogenicity test was performed on the storage roots of the cultivar Beniharuka (Kai et al., 2010). Three replicates of storage roots were used for each isolate. The roots were rinsed three times with SDW after dipping them in 4% sodium hypochlorite for 1 min. The storage roots were transferred onto sterilized clean boxes with moisture and wounded using a sterile needle. The wounds were inoculated with a 10 μL spore suspension (1 × 10^6^ spores/mL). The infected roots were incubated for 7 d at 25 °C in the dark. Then, the lesion lengths were measured to select a representative isolate showing the highest severity for phenotypic evaluation.

### 2.2 Plant materials

Ninety-six sweet potato genotypes, including cultivars, landraces, and lines, collected from the Bioenergy Crop Research Institute (Muan, Korea) were used for GWAS to dissect the genetic basis of Fusarium root rot. Among them, 45 genotypes originated from South Korea, 21 from Japan, 11 from China, 16 from Taiwan, 1 from Indonesia, 1 from New Zealand, and 1 from Ethiopia (**Supplementary Table 1**). The 96 genotypes have been carefully selected based on their parents from other countries and their quantitative traits such as response to Fusarium root rot, agronomic traits, starch, sugar contents, and polyphenol.

All genotypes (n = 96) were planted in the Bioenergy Crop Research Institute field in 2021 and 2022. Then, the storage roots were harvested after 120 d and stored at 13 °C and 85–90% relative humidity for phenotypic evaluation.

### 2.3 Phenotypic evaluation for Fusarium root rot

To evaluate resistance to Fusarium root rot, the storage roots of 96 genotypes were inoculated with a spore suspension. The *F. solani* isolate SPL21019 was incubated for 7 d, and the concentration of the suspension was adjusted to 1 × 10^7^ spores/mL. Three roots per genotype were soaked in 4% sodium hypochlorite for 1 min and rinsed three times with SDW. After drying, the roots were wounded with a sterilized needle and then inoculated with 10 μL of the spore suspension. The roots were relocated onto a plastic mesh platform in sterilized boxes with moisture and incubated at 25 °C for 14 d. The lesion diameters of the diseased roots were measured, and their average value was used for further analysis.

### 2.4 DNA extraction and genotyping

Fresh leaves of the genotypes were sampled and ground into a powder using liquid nitrogen. Genomic DNA was extracted using the modified cetyltrimethylammonium bromide (CTAB) method described by Kim and Hamada (2005). The quality and quantity of genomic DNA were confirmed using a NanoDrop 2000 spectrophotometer and electrophoresis on 1.0% agarose gel. GBS was performed following a previously reported method (Elshire et al., 2011). The genomic DNA was digested using the *ApeK*I enzyme (New England Biolabs, Ipswich, MA, USA) at 75 °C for 3 h. The DNA fragments were then ligated to adapters, with different barcodes and common adapters assigned to each sample. Amplified DNA samples were pooled and purified using the PCR Purification system (Biofact, Korea). The resulting GBS libraries were sequenced using an Illumina HiSeqXten system (Illumina, San Diego, CA, USA). After sequencing the samples, barcode sequences were eliminated using Ipyrad (v.0.9.8, Eaton and Overcast, 2020), and short reads were trimmed using Trimmomatic (v.0.38, Bolger et al., 2014). Sorted reads were aligned to the sweet potato reference genome (https://sweetpotao.com/; Yang et al., 2017) using BWA-MEM (v.0.7.17, Li, 2013). Samtools (v.1.15, Li et al., 2009) was used to generate the BAM format and reorder the reads. PCR duplicates were removed using Picard (v.2.25.6; https://broadinstitute.github.io/picard/), and SNP calling was performed using HaplotypeCaller- and CombineGVCFs-GATK (v.4.2, McKenna et al., 2010). Raw SNPs were filtered using VCF tools (Danecek et al., 2011) with the following parameters: –min-alleles 2 –max-alleles 2 –minDP 5 –minGQ 20 –max-missing 0.4 –maf 0.05. The output SNP set was used for structural and GWAS analyses. The SNP density plots were visualized using the R package *CMplot* (v.4.3.0, Yin et al., 2021).

### 2.5 Population structure analysis

After further filtering for missing rates < 20%, a subset of 13,153 SNPs was used for population structure analysis. Structural analysis of the population was conducted using the Bayesian Markov Chain Monte Carlo model in STRUCTURE (v.2.3.4, Pritchard et al., 2000). The *K* value (number of subpopulations), designated from 1 to 10, was used for 5,000 burn-ins and 50,000 run-lengths with three replications. The STRUCTURE results were then used to obtain the maximum peak *K* value using STRUCTURE HARVESTER (v0.6.94, Earl and vonHoldt, 2012). A phylogenetic tree was constructed using the maximum composite likelihood method with 5,000 repeats of the NJ method in MEGA-X (Saitou and Nei, 1987).

### 2.6 Genome-wide association study

After filtering, 44,255 SNPs were used for the GWAS analysis. GWAS was conducted for the phenotypic data on two (2021 and 2022) and combined (both) years using Fixed and random model Circulating Probability Unification (FarmCPU) and Bayesian information and the linkage disequilibrium iteratively nested keyway (BLINK) of GAPIT (Liu et al., 2016; Huang et al., 2019; Wang and Zhang, 2021). The GWAS results were visualized using Manhattan plots drawn with R package *qqman* (Turner, 2014). Bonferroni correction was calculated as − log_10_ (0.05/*n*) and − log_10_ (1/*n*), where *n* is the number of SNPs used in the GWAS.

### 2.7 Real-time quantitative reverse transcription PCR for candidate genes

Real-time quantitative reverse transcription PCR (qRT-PCR) was performed with three biological replicates to analyze the expression of candidate genes identified using GWAS. Three storage roots of two genotypes, Geonhwangmi (Lee et al., 2016) and Beniharuka, were used to compare gene expression at 0, 1, 3, 6, 12, and 24 h post-inoculation with 1 × 10^7^ spores/mL of SPL21019 isolate. Total RNA was extracted from the storage roots at different time points after inoculation using a modified CTAB method (Kim and Hamada, 2005). Then, the cDNA was synthesized using the PrimeScript II 1^st^ Strand cDNA Synthesis Kit (TaKaRa, Japan). RT-PCR was performed to analyze gene expression using iTaq Universal SYBR Green Supermix (Bio-Rad, USA) on a Bio-Rad CFX Opus 96 Real-Time PCR System (Bio-Rad, USA). The amplification reaction conditions were: pre-denaturation at 95 °C for 30 s, followed by 40 cycles of 3 s at 95 °C, and 20 s at 58 °C. The melt curve analysis was performed under the following conditions: a slow increase in temperature by 0.5 °C every 5 s from 60 °C to 95 °C, with constant fluorescence measurement. The gene-specific primers used in this study were designed using Primer 3 (v.4.1.0, Untergasser et al., 2012) and are listed in **Supplementary Table 2**. Data analysis was performed using CFX Maestro Software (v.2.3, Bio-Rad, USA) with 2^-ΔΔ^*^Ct^* method. *Ubiquitin extension protein* (*UBI*) was used as a reference gene for normalization (5′-TCG ACA ATG TGA AGG CAA AG-3′ and 5′-CTT GAT CTT CTT CGG CTT GG-3′; Park et al., 2012).

### 2.8 Statistical analysis

The phenotypic data in 2021 and 2022 were analyzed using a one-way analysis of variance (ANOVA). Correlation analysis between phenotypic data obtained in 2021 and 2022 was performed using the R package. Significant differences in lesion lengths by pathogenicity tests with seven isolates were analyzed using Duncan’s multiple range test (DMRT). The statistical differences in expression of the candidate genes in Geonhwangmi and Beniharuka at every time point after inoculation were assessed using Student’s t-test. All statistical analyses and descriptive statistics were performed using the R package (R Core Team, 2017).

## 3 Results

### 3.1 Pathogen isolation and pathogenic analysis

**Figure 1:**
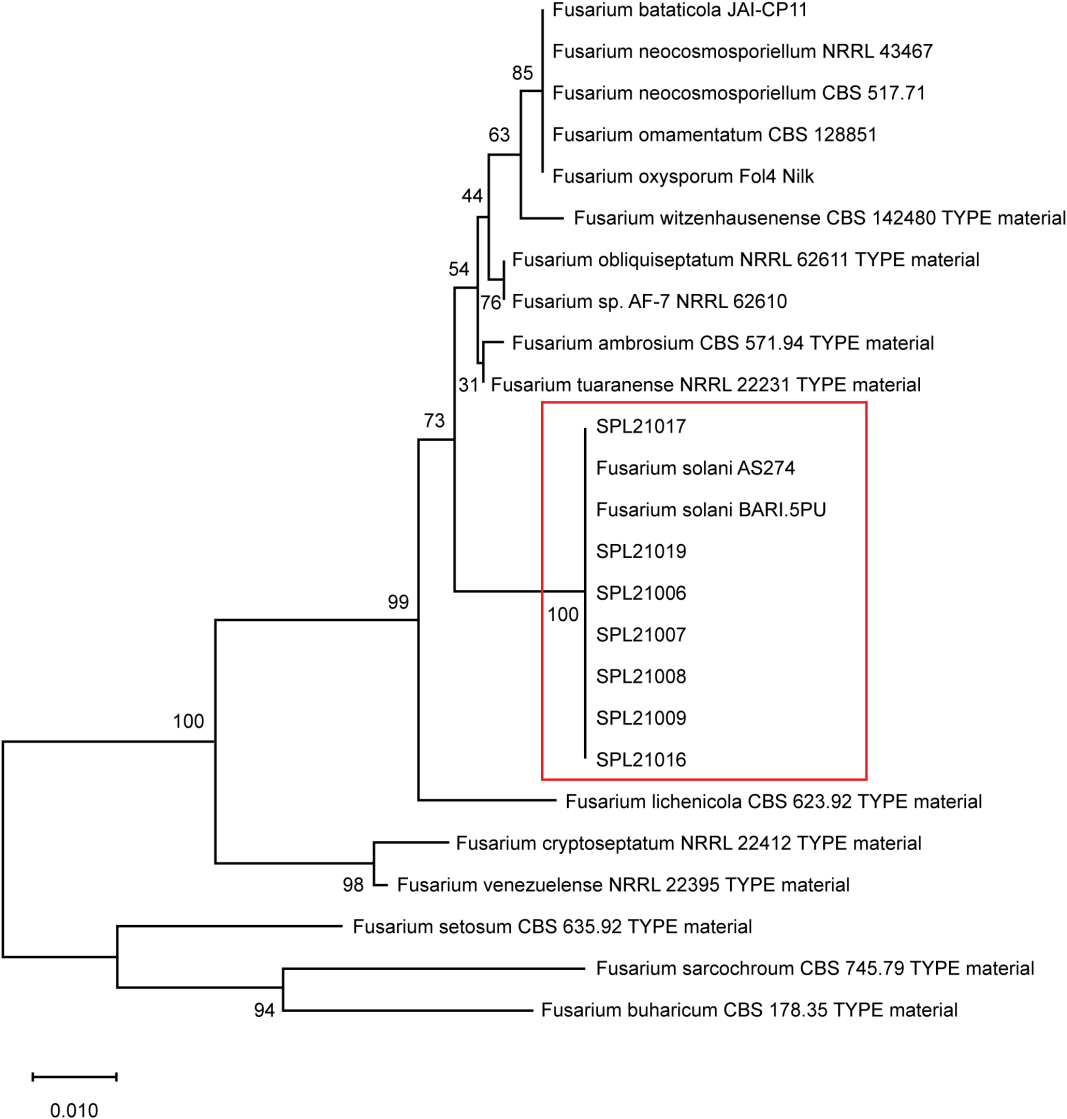
Phylogenetic tree of isolates based on the ITS region of rDNA sequences. Bootstrap support values for 100 replicates by the neighbor-joining method and MEGA X analysis.

**Figure 2:**
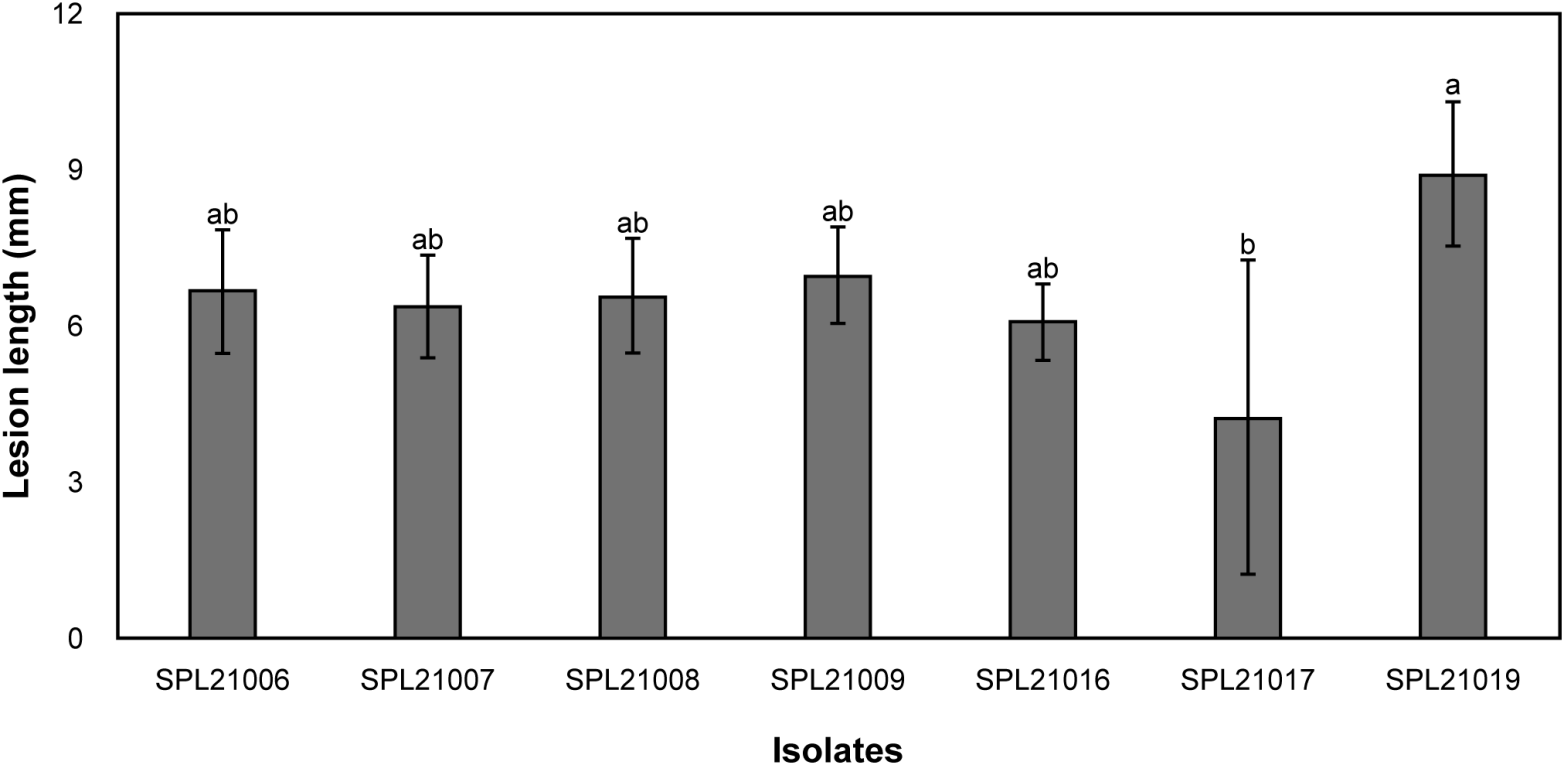
Comparisons in lesion length (mm) resulting from pathogenicity test among the isolates. Vertical bars represent the standard deviation of three biological replicates. Columns with different letters indicate treatment means that are significantly different according to Duncan’s multiple range test (*P* < 0.05).

Hollowed lesions were discovered on the surface of the storage roots that reached the periderm. After phylogenetic analysis of the ITS-rDNA sequences, seven isolates revealed similarities of 99–100% and a high bootstrap value of 100% with reference isolates of *F. solani* (AS274 and BARI.5PU) (**Figure 1**). The seven isolates produced an orange color in the agar and white colonies on PDA for 7 d at 25 °C. In the pathogenicity tests, Fusarium root rot symptoms were observed on the surface of the roots, and the pathogens were re-isolated from the lesions. The lesion lengths of the seven isolates measured using the pathogenicity test are shown in **Figure 2**. The lesion length of SPL21019 (NCBI accession number MZ930186) was significantly longer than that of the other isolates, and SPL21019 was selected as the representative isolate.

### 3.2 Phenotypic evaluation of Fusarium root rot resistance

**Figure 3:**
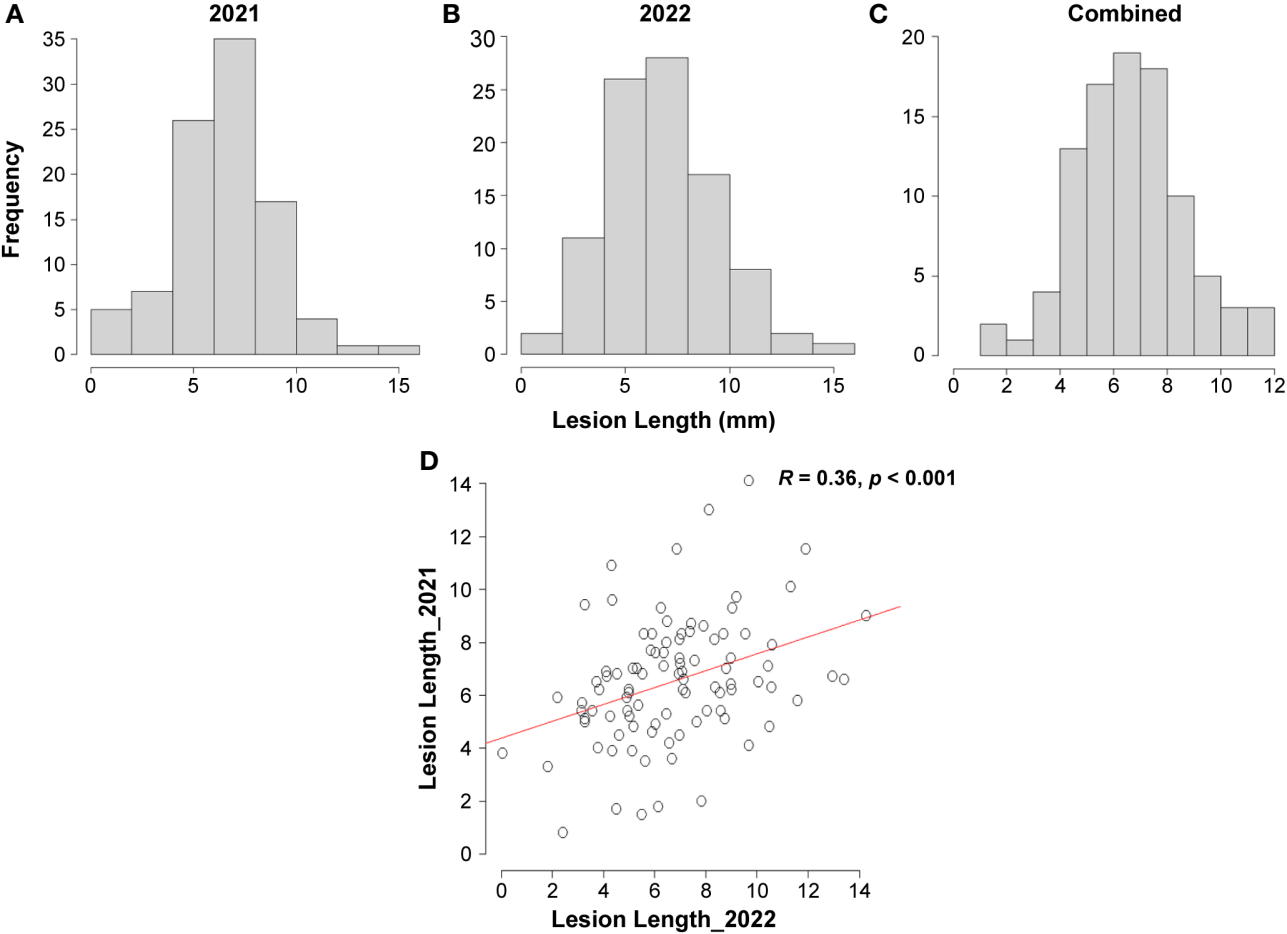
Distribution of lesion length observed 14 d after inoculation among 96 genotypes. **(A)** 2021, **(B)** 2022, **(C)** combined years, **(D)** Pearson’s correlation between the two years.

To investigate the variation in Fusarium root rot resistance in the 96 genotypes, the lesion length was investigated 14 d after inoculation in 2021 and 2022. The phenotypic evaluation revealed a normal distribution for both years, separately and combined (**Figures 3A–C**). Correlation analysis showed significantly positive correlations between the two years (*R* = 0.36, *p* < 0.001) (**Figure 3D**). The lesion length ranged from 0.80 mm (Geonhwangmi) to 14.06 mm (Beniharuka) with a mean of 6.53 mm in 2021 and from 0.05 mm (Kanto 48) to 14.27 mm (Suwon 109) with a mean of 6.73 mm in 2022. In combined data of the years, the lesion length ranged from 1.61 mm (Geonhwangmi) to 11.89 mm (Beniharuka), with a mean of 6.63 mm. Geonhwangmi, which was relatively resistant, showed lesion lengths of 0.80, 2.42, and 1.61 mm of lesion length, and Beniharuka, which was relatively susceptible, showed lesion lengths of 14.06, 9.68, and 11.89 mm in 2021, 2022, and the combined years, respectively. The coefficients of variance (CV) for 2021, 2022, and the combined years were 35.5, 39.1, and 30.5%, respectively (**Supplementary Table 3**). The ANOVA results revealed highly significant differences (*p* < 0.001) across the genotypes (G) and genotype × year (G × Y) interactions but no significant differences between the two years (Y) (**Table 1**).

**Table 1:** Analysis of variance (ANOVA) for Fusarium root rot in the GWAS genotypes.

Source	Df	MS	F
Genotype (G)	95	26.07	9.58^***^
Year (Y)	1	0.99	0.36
G × Y	94	11.86	4.36^***^
Residuals	378	2.72	

*** indicates significance at P ≤ 0.001.

### 3.3 Resequencing of sweet potato genotype resources and SNP calling

**Figure 4:**
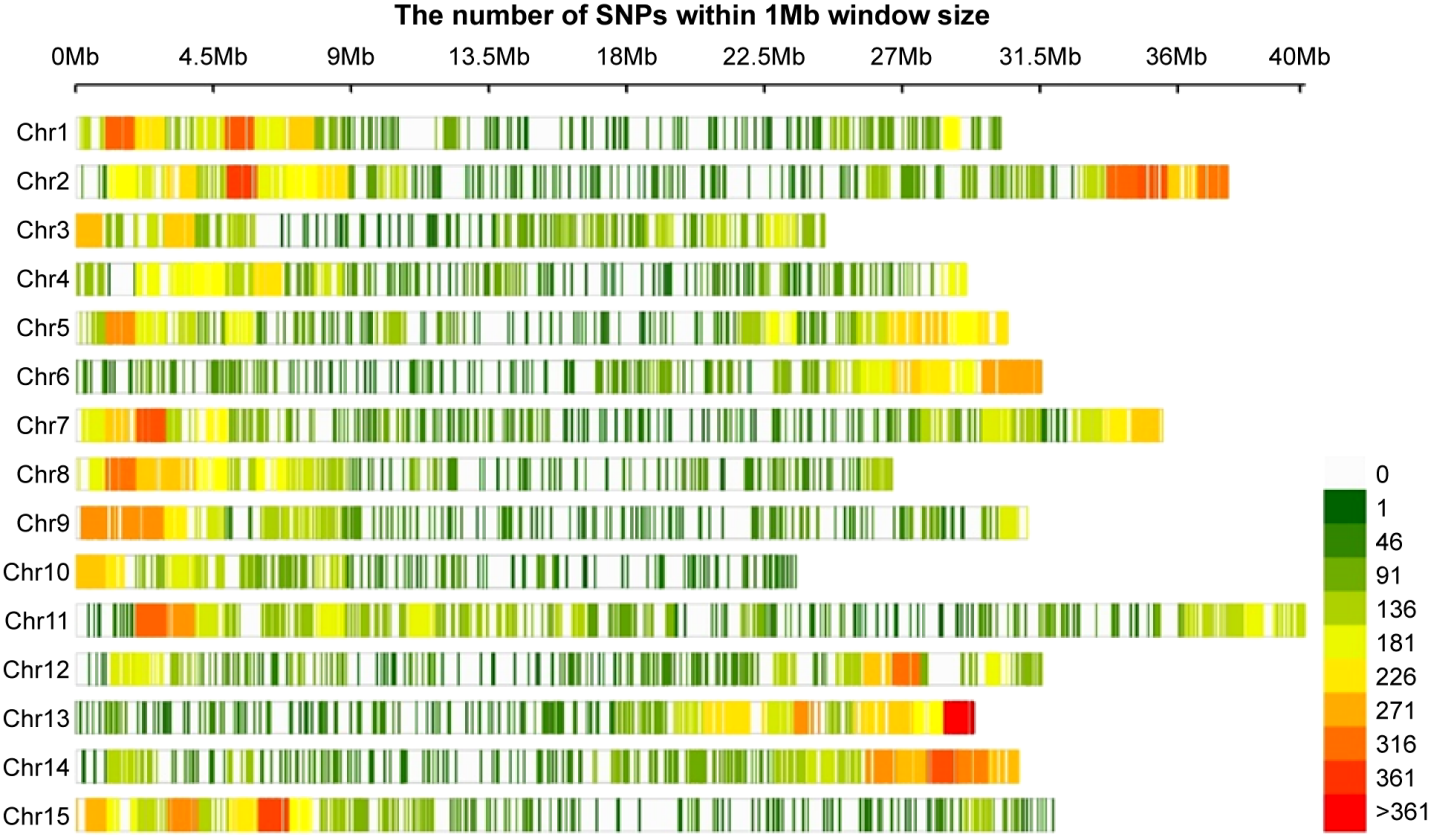
The density plot of SNPs across 15 sweet potato chromosomes.

Sequencing was performed after constructing a GBS library of the 96 genotypes. The sequenced raw reads ranged from 39,418 bp (Jangseong jaerae; 5,952,118 bp) to 143,211,472 bp (Benihayato; 21,624,932,272 bp), with an average of 6,901,756 bp. After trimming an average of 27.8% reads per sample using Trimmomatic, 5,413,595 (768,201,397 bp) reads were mapped to the reference genome Taizhong 6. The average mapping rate was 89.8% (**Supplementary Table 4**). A total of 1,429,321 SNPs were called (**Supplementary Table 4**), and 44,255 SNPs distributed across all 15 chromosomes of the sweet potato genome were identified after filtration (**Figure 4**). The SNPs ranged from 1,729 (chromosome 9) to 4,321 (chromosome 1), with an average of 2,950 SNP numbers per chromosome. A total of 44,255 SNPs were selected for GWAS analysis.

### 3.4 Population structure analysis

**Figure 5:**
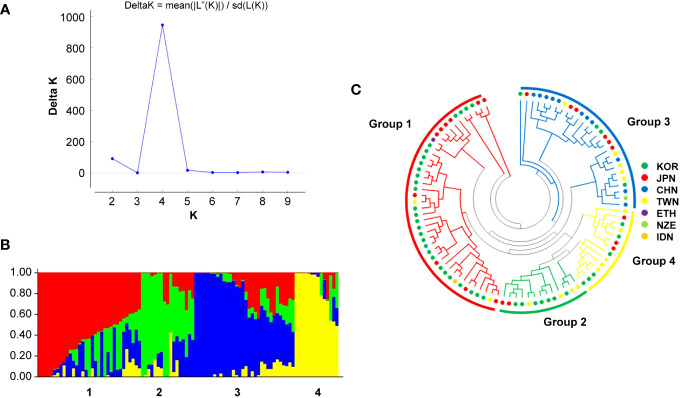
Population structure and phylogenetic analysis of 96 sweet potato genotypes. **(A)** Delta K graph calculated using the Evanno method and **(B)** optimal population structure (delta K = 4). **(C)** Phylogenetic analysis using the neighbor-joining method.

The population was divided into four groups according to the optimum *K* value of 4 obtained in the Bayesian model using the STRUCTURE software and phylogenetic analysis (**Figure 5**). Group 1 contained 33 recently developed relatively resistant cultivars from South Korea; some cultivars in this group have the same parent (Kokei 14) in their pedigree. Group 2 included 17 genotypes, primarily breeding lines or cultivars developed before 2000 in South Korea and Taiwan. Group 3 comprised 32 genotypes primarily from China, and Group 4 contained 14 genotypes primarily from South Korea and Taiwan.

### 3.5 GWAS and identification of candidate genes

**Figure 6:**
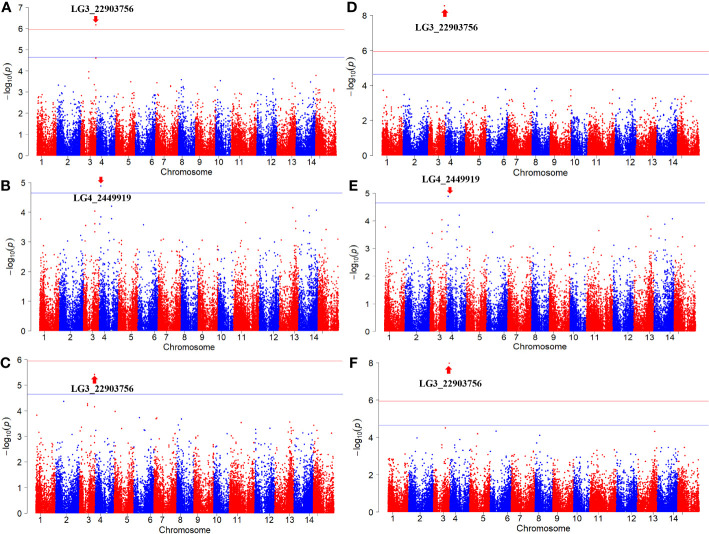
Manhattan plots of results in GWAS analysis. **(A, D)** 2021, **(B, E)** 2022, **(C, F)** combined years. **(A–C)** FarmCPU, **(D–F)** BLINK. The horizontal lines indicate the Bonferroni-corrected threshold. Red lines indicate - log_10_ (*P*) = 5.95 and blue lines indicate - log_10_ (*P*) = 4.64.

**Figure 7:**
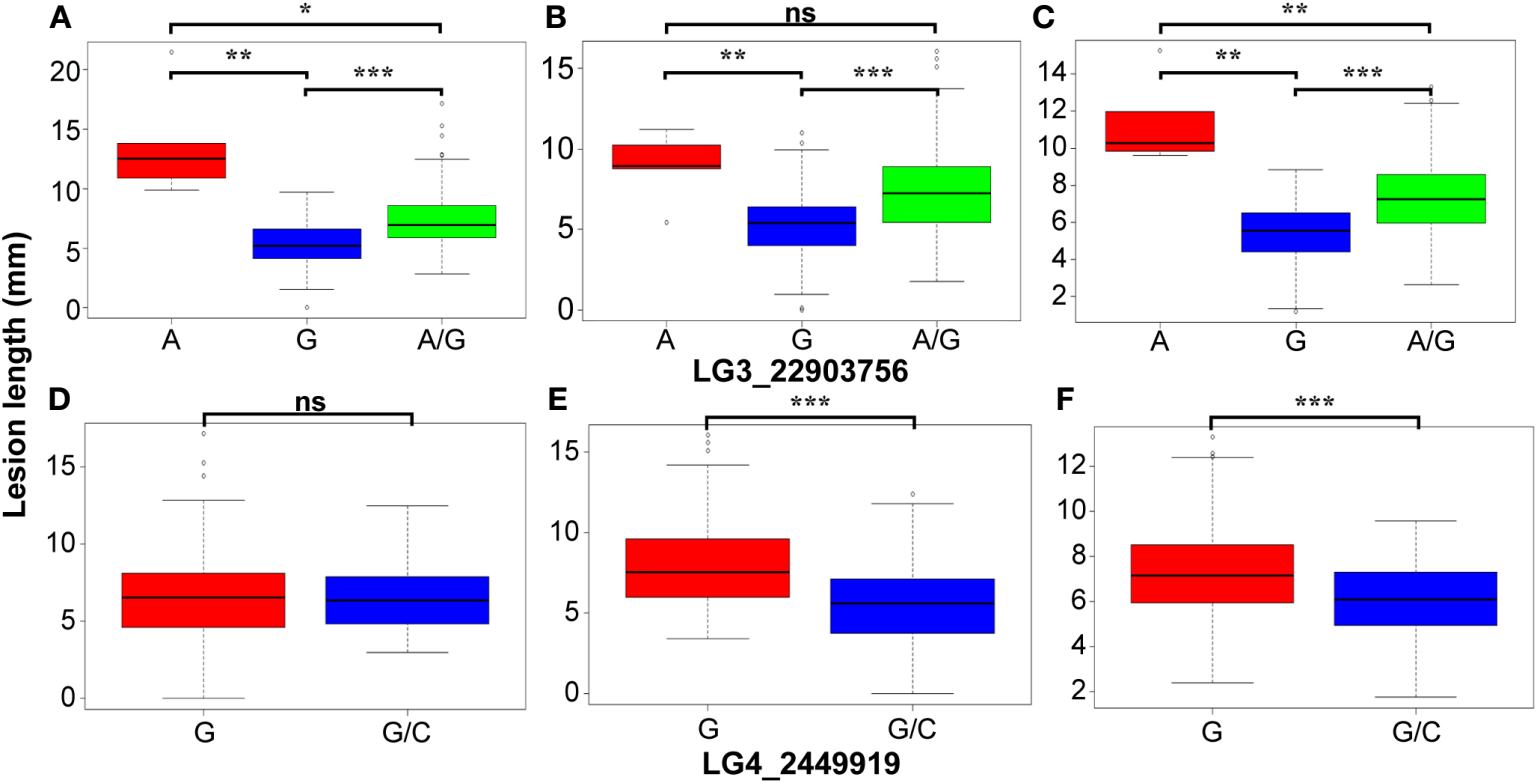
Comparison of the lesion length in genotypes with different alleles of two significant SNPs. **(A, D)** 2021, **(B, E)** 2022, **(C, F)** combined years. ns, *, **, *** indicate not significance, significance at *P* ≤ 0.05, 0.001 and 0.0001, respectively.

The GWAS was conducted on Fusarium root rot resistance based on 44,255 SNPs using the FarmCPU and BLINK models. These methods identified significant SNPs on chromosome 3 (LG3_22903756) in 2021 and the combined years and on chromosome 4 (LG4_2449919) in 2022 with −log_10_ (*p*) > 5.95 and 4.65, respectively, as shown in the Manhattan plots (**Figure 6**; **Table 2**). Moreover, the genotypes carrying the SNP LG3_22903756-A allele exhibited a significantly higher average lesion length than those carrying the SNP LG3_22903756-G allele in 2021, 2022, and the combined years. The differences in lesion length between SNP LG4_2449919-G and LG4_2449919-G/C alleles were significant in 2022 and the combined years (**Figure 7**). LG3_22903756 was found in *g12495*, and 23 genes encoding functional proteins were identified within the 100 kb flanking regions of LG3_22903756. Of these genes, five genes encoding receptor-like protein kinases, late blight resistance protein homologs, and serine/threonine protein kinases were identified as potential candidate genes. LG4_2449919 was found in *g13128*. Twenty-nine functional protein-encoding genes were identified within the 100 kb flanking regions of LG4_2449919; of these, six genes involved in disease resistance and encoding inorganic phosphate transporters, ethylene-responsive transcription factor, WAT1 (walls are thin 1)-related protein, and ethylene receptor homologs were identified as putative candidate genes (**Table 3**).

**Table 2:** SNPs detected by GWAS analysis for Fusarium root rot resistance.

Model	Year	SNP	Chromosome	Position	- log_10_ (*P*)	MAF	FDR-adjusted *P* values	effect
FarmCPU	2021	LG3_22903756	3	22903756	6.17	0.4	0.030152	-2.57
	2022	LG4_2449919	4	2449919	4.88	0.3	0.584259	-2.83
	Avg.	LG3_22903756	3	22903756	5.42	0.4	0.168593	-2.21
BLINK	2021	LG3_22903756	3	22903756	8.55	0.4	0.000123	-2.57
	2022	LG4_2449919	4	2449919	4.79	0.3	0.711537	-2.82
	Avg.	LG3_22903756	3	22903756	8.27	0.4	0.000236	-2.35

**Table 3:** Candidate genes for Fusarium root rot resistance.

Chr.	Start (bp)	End (bp)	Gene ID	Functional Annotation
3	22,860,497	22,887,078	g12492	probable receptor-like protein kinase At1g67000 isoform X2 [Nicotiana tomentosiformis]
3	22,888,555	22,893,108	g12493	putative late blight resistance protein homolog R1B-14 [Nicotiana sylvestris]
3	22,894,510	22,898,718	g12494	probable receptor-like protein kinase At1g67000-like [Solanum tuberosum]
3	22,902,113	22,908,469	g12495	unknown
3	22,916,792	22,920,968	g12497	probable serine/threonine-protein kinase At1g18390 isoform X1 [Nicotiana tomentosiformis]
4	2,449,610	2,451,982	g13128	inorganic phosphate transporter 1-4-like [Sesamum indicum]
4	2,474,885	2,476,112	g13132	ethylene-responsive transcription factor 1B [Sesamum indicum]
4	2,487,357	2,488,048	g13133	ethylene-responsive transcription factor ERF098-like [Nicotiana sylvestris]
4	2,488,381	2,489,472	g13134	ethylene-responsive transcription factor 14-like [Solanum tuberosum]
4	2,503,123	2,505,352	g13136	WAT1-related protein At4g08300-like [Solanum lycopersicum]
4	2,542,078	2,543,372	g13143	ethylene receptor homolog [Nicotiana tabacum]

### 3.6 Expression analysis of candidate genes

**Figure 8:**
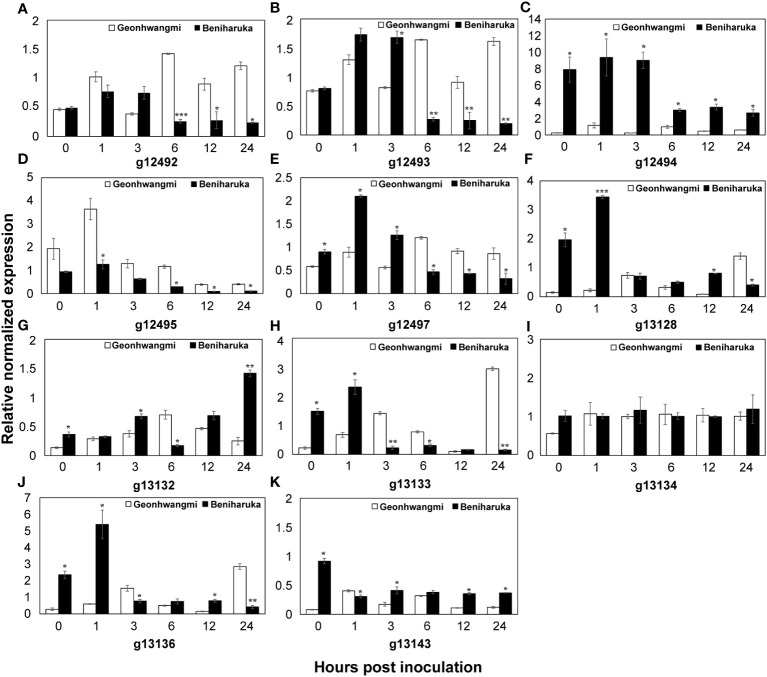
qRT-PCR expression analysis of the candidate genes. The storage roots were analyzed at 0, 1, 3, 6, 12, and 24 h after inoculation of SPL21019 in Geonhwangmi (resistant) and Beniharuka (susceptible). The relative normalized expression of each candidate gene **(A–K)** was determined. Vertical bars mean the standard error of the means. *, **, *** mean significance at *P* ≤ 0.05, 0.001, and 0.0001.

qRT-PCR was conducted to verify the expression patterns of 11 candidate genes involved in Fusarium root rot resistance in Geonhwangmi (resistant) and Beniharuka (susceptible). The expression of *g12492* and *g12493* increased at 1 and 3 h post-inoculation in both cultivars and showed significantly high levels in Geonhwangmi at 6 h post-inoculation (**Figures 8A, B**). In addition, the expression of *g12495* and *g12497* peaked at 1 and 6 h after inoculation in Geonhwangmi (**Figures 8D, E**). The expression of *g12494, g13128*, and *g13136* peaked at 1 h post-inoculation in Beniharuka (**Figures 8C, F, J**). At 24 h post-inoculation, *g13132* peaked in Beniharuka, whereas *g13133* peaked in Geonhwangmi (**Figures 8G, H**). Two genes, *g13134* and *g13143*, were regularly expressed at every time point in both cultivars (**Figures 8I, K**).

## 4 Discussion

Fusarium root rot, caused by *F. solani* in the soil, has been reported as a major post-harvest disease in sweet potatoes (Scruggs and Quesada-Ocampo, 2016). To develop resistant cultivars, the QTL and genes associated with Fusarium root rot resistance have to be identified (Yang et al., 2017). However, the genetic study of sweet potatoes has been challenging due to the homologous and hexaploid genome. With the development of next-generation sequencing technology, the reference sweet potato diploid or hexaploid genomes were sequenced. In addition, GBS and restriction site-associated DNA sequencing have enabled the production of markers and the construction of a high-density genetic map (Yamamoto et al., 2020). Recently, GWAS for weevil resistance, nematode resistance, anthocyanin contents, starch contents, and root flesh color have been conducted with sequenced reference genomes in sweet potatoes (Hirakawa et al., 2015; Okada et al., 2019; Haque et al., 2020; Liu et al., 2022b; Obata et al., 2022; Nie et al., 2023). To the best of our knowledge, this is the first study to identify genomic regions and genes underlying Fusarium root rot resistance in sweet potatoes using GWAS.

Frequencies of genotypes showed a normal distribution according to lesion length in 2021 and 2022, as well as in the combined years. The two years showed a significantly positive correlation, indicating that the resistance test used in this study is a reliable method to evaluate resistance to Fusarium root rot. The ANOVA showed that genotype (G) and genotype by year (G × Y) significantly affected lesion length, but the years had no significant effects. This result suggests that most phenotypic variations in the association mapping population are genetically controlled, not environmentally. The disease index frequencies of F_1_ progenies were abnormal, and only 52.6–57.0% of the phenotypic variation was explained by seven QTL by Ma et al. (2020); however, two significant SNPs explained 73.3–85.7% of the phenotypic variation in this study.

Structural analysis was used to assess the population structure of 96 genotypes that were mainly from Asia (South Korea, China, Japan, and Taiwan). The population was divided into four groups using an admixture model and phylogenetic analysis, and the results did not match the geographic collection location, as seen in studies by Su et al. (2017) and Wadl et al. (2018). These results may be explained by the exchange of genetic resources among countries and the resulting recombination of resources.

Although GWAS is a powerful approach for the identification of novel genes with high-density SNP genotyping, its results are affected by several factors such as population, SNP coverage, and phenotypic data, generating false-positive associations, which is a Type I error (Rafalski, 2010; Zahid et al., 2022). However, the results of eQTL mapping with 86 sweet potato accessions showed identified eQTL co-located with QTL involved in anthocyanin accumulation, indicating sufficient size for identifying significance (Zhang et al., 2020). In addition, GWAS with 66 genotypes, including 42 from Korea and 12 from Japan, showed seven sugar and starch metabolism-related genes identified from 39,424 high-quality SNPs (Nie et al., 2023). In our study, the population size is relatively small, with almost half of the genotypes originating from South Korea. However, we selected the genotypes, which are progenies of different parents from other countries developed since 1961, based on the response to Fusarium root rot, and used 44,255 high-quality SNPs evenly distributed across the chromosomes. In addition, to reduce the false-positive associations, we set the threshold by Bonferroni correction and used multi-locus models such as FarmCPU and BLINK. The Bonferroni correction threshold has been widely used to control false positives (Zhang et al., 2019). Furthermore, FarmCPU and BLINK models are multivariate methods to reduce false positives and increase statistical power (Wang and Zhang, 2021). The FarmCPU model was developed to prevent model over-fitting and control false positives by testing the markers as covariates in a fixed effect model and optimizing the covariate markers in a random effect model, as a single locus model can miss the important markers (Liu et al., 2016). The BLINK model was improved from FarmCPU by ignoring the assumption that important genes are evenly distributed across the genome with increasing statistical power (Merrick et al., 2021). The results of the GWAS showed that the two SNPs were markedly related to Fusarium root rot resistance. The mean lesion length significantly increased in genotypes carrying the LG3_22903756-A allele compared to those carrying the LG3_22903756-G allele in 2021, 2022, and the combined years. Although genotypes with LG4_2449919-G and LG4_2449919-G/C alleles showed significant differences in lesion length, no genotype with homozygous alleles for LG4_2449919-C was identified. These results indicate that the SNP LG3_22903756 is highly related to Fusarium root rot resistance genes and may be converted into molecular markers for selecting Fusarium root rot-resistant sweet potato cultivars in breeding programs. LG3_22903756 and LG4_2449919 are located in the intergenic regions of *g12495* and *g13128*, respectively. However, the function of *g12495* has not been explored, warranting further studies to understand its role in Fusarium root rot resistance.

Candidate genes encoding receptor-like protein kinases, late blight resistance proteins, and serine/threonine protein kinases have been identified as resistance (R) genes that recognize both general ligands and specific pathogens (Goff and Ramonell, 2007). Ethylene Response Factors (ERFs) include genes encoding ethylene-responsive transcription factors and ethylene receptors that perceive the AGCCGCC motif (the GCC box) to control the response to pathogens (Müller and Munné-Bosch, 2015). A gene encoding a WAT1-related protein is required for secondary cell-wall deposition, thereby conferring resistance to vascular pathogens (Denancé et al., 2013).

The expression of the identified genes in Geonhwangmi (resistant) and Beniharuka (susceptible) cultivars were analyzed to predict their function in Fusarium root rot resistance. Genes *g12492* (probable receptor-like protein kinase *At1g67000* isoform X2) and *g12494* (probable receptor-like protein kinase *At1g67000*-like) showed contrasting expression patterns between the resistant and susceptible cultivars at all tested time points. The expression of *g12497* (probable serine/threonine-protein kinase *At1g18390* isoform X1) was low at 0, 1 and 3 h, and it increased significantly at 6 h post-inoculation in Geonhwangmi. Receptor-like kinases (RLKs), which belong to a large superfamily, are involved in various plant responses to hormones and pathogens (Goff and Ramonell, 2007). Most plant RLKs are located in the plasma membrane. The N-terminal extracellular domain of RLKs identifies internal and external ligands, whereas the cytoplasmic serine/threonine protein kinase domain phosphorylates them, initiating downstream signaling (Gish and Clark, 2011). *At1g67000* and *At1g18390* encode the leaf rust 10 disease-resistance locus receptor-like protein kinase-like (*LRK10*) and are located at the leaf rust (*Lr*) *10* resistance site in wheat (Shin et al., 2015; Ma et al., 2022). Overexpression of *Lr10* results in enhanced resistance with complete prevention of rust sporulation (Feuillet et al., 2003). Although *g12492* and *g12494* are homologous with *At1g67000*, they showed 81.7% sequence similarity when aligned on the reference genome. These data indicate that the observed differences in sequences could be associated with the opposite expression patterns between Geonhwangmi and Beniharuka. *g12492*, highly expressed in resistant cultivars, may confer resistance to Fusarium root rot in sweet potatoes.

*g12493* (putative late blight resistance protein homolog R1B-14) increased at all tested time points in Geonhwangmi but decreased at 6 h post-inoculation in Beniharuka, suggesting *g12493* is a key gene in Fusarium root rot resistance in sweet potatoes. The late blight resistance *R1* gene contributes to the resistance to specific races of pathogens and is stacked in potato varieties (Wastie, 1991). The *R1* gene is in a hotspot for resistance to pathogens and viruses, such as *Phytophthora infestans* (Ritter et al., 1991; Leonards-Schippers et al., 1992; De Jong et al., 1997).

*g12495* included the significant SNP LG3_22903756 and was expressed at a higher level in Geonhwangmi, annotated as a gene by AUGUSTUS (Stanke et al., 2006). The BLAST results showed more than 95% identity with uncharacterized non-coding (nc) RNA of *I. triloba*, which was predicted by automated computational analysis with Gnomon (Souvorov et al., 2010; Wu et al., 2018). The ncRNAs have also been reported to regulate the immune response in plant signaling pathways; therefore, *g12495* should be investigated in future studies (Song et al., 2021).

*g13128* (inorganic phosphate transporter 1-4-like), possessing the significant SNP LG4_2449919, was highly expressed in the susceptible cultivars. The line overexpressing *OsPT8*, which encodes a phosphate transporter protein, was more susceptible to pathogens in rice, indicating that this gene may negatively regulate disease responses, consistent with the results of our study (Dong et al., 2019). ERFs act as transcriptional activators or repressors and regulate plant stress resistance (Thirugnanasambantham et al., 2015). *g13132* (ethylene-responsive transcription factor 1 B) was expressed at a low level and increased at 24 h post-inoculation in the susceptible cultivar. *ERF1* functions as a component of the ethylene and jasmonate pathways and regulates the expression of ethylene/jasmonate-dependent defense genes (Lorenzo et al., 2003). However, *ERF1* overexpression does not increase the tolerance of *Pseudomonas syringae* in tomato DC3000, indicating that this gene may play a negative role in sweet potatoes (Berrocal-Lobo et al., 2002). In contrast, the expression of *g13133* (ethylene-responsive transcription factor ERF098-like) was high early post-inoculation and decreased in the susceptible cultivar but increased late post-inoculation in the resistant cultivar. *AtERF98* belongs to Group IX of the ERF family, which has often been associated with defensive gene expression against pathogen infections (Nakano et al., 2006). In group IX, the ascorbate synthesis genes are regulated by *AtERF98*, which enhances salt tolerance, although its function in biotic stress has not been proven (Zhang et al., 2012). *ERF098* may be a late response gene for pathogenic infections in sweet potatoes. *g13134* (ethylene-responsive transcription factor 14-like) and *g13143* (ethylene receptor homolog) were expressed at low levels in both cultivars, indicating that these genes may not be related to Fusarium root rot resistance.

*g13136* (WAT1-related protein At4g08300-like) was significantly upregulated in the susceptible cultivar early post-inoculation. The *wat1* mutant shows resistance to all vascular pathogens compared to the wild type in *Arabidopsis thaliana*, and the roots contain higher amounts of salicylic acid (SA) and lower levels of indole metabolites (Denancé et al., 2013). The SA levels increase upon infection with the soil-borne vascular pathogen *Verticillium longisporum*, inhibiting *F. oxysporum* cells and preventing Fusarium wilt in potatoes (Ratzinger et al., 2009; Li et al., 2022). These results indicate that higher expression of WAT1-related proteins may inhibit the expression of SA metabolites and allow pathogen infection. Combined with the expression analysis of candidate genes, the upregulated (*g12492, g12493, g12495, g12497*, and *g13133*) and downregulated (*g12494, g13128, g13132*, and *g13136*) genes may be involved in resistance to Fusarium root rot in sweet potatoes.

This study was conducted to identify candidate QTL and genes for Fusarium root rot resistance in sweet potatoes. However, the sample size used for the GWAS was relatively small, and genotypes may not fully represent the genetic diversity in sweet potato populations. Furthermore, the phenotypic evaluation was performed under controlled conditions to reduce environmental variables. Further studies with sufficiently large sample sizes and field tests may enhance the understanding of Fusarium root rot resistance in sweet potatoes.

In conclusion, the present study demonstrates a GWAS to assess Fusarium root rot resistance based on 44,255 SNPs in 96 sweet potato genotypes. Phenotypic data indicated significant variation between genotypes and identified multiple genes associated with Fusarium root rot resistance. Two SNPs were identified in 2021, 2022, and the combined years by GWAS. The expression analysis of the genes in flanking region of the identified QTL showed nine genes with altered expression, indicating their association with Fusarium root rot resistance in sweet potatoes. Nevertheless, further molecular analyses of these genes may aid in better understanding the mechanisms of Fusarium root rot resistance. Additionally, the resistance to diseases of *Fusarium* spp. may be enhanced by identifying and characterizing candidate genes conserved in other plants. The significant SNPs can be incorporated into the future marker-assisted selection to develop resistant sweet potato cultivars.

## Data availability statement

The datasets presented in this study can be found in online repositories. The names of the repository/repositories and accession number(s) can be found below: NCBI accession number PRJNA849767.

## Author contributions

TK designed the experiments and wrote the manuscript. TK and SK collected samples with disease and performed pathogenicity tests on isolated pathogens. SK, WP, KW, J-DL, and HJ drafted the work or revised it critically for important intellectual content. MC, YL, H-UL, KHL, S-SN, and KL provided approval for the publication of the content All authors contributed to the article and approved the submitted version.
