# Supplementary material for: Genome-wide association study to identify novel loci and genes for Fusarium root rot resistance in sweet potato using genotyping-by-sequencing
Source: Front Plant Sci. 2023 Oct 4;14:1251157. doi: 10.3389/fpls.2023.1251157 (PMC10584150; doi:10.3389/fpls.2023.1251157)
Supplement: Supplementary file 1 [file Table_1.docx]

Supplementary Material

Genome-wide association study to identify novel loci and genes for Fusarium root rot resistance in sweet potato using genotyping-by-sequencing

Tae Hwa Kim*, Sujung Kim, Won Park, Koan Sik Woo, Keunpyo Lee, Mi Nam Chung, Young Hoon Lee, Hyeong-Un Lee, Kyo Hwui Lee, Sang-Sik Nam, Hyun Jo, Jeong-Dong Lee

*** Correspondence:** Tae Hwa Kim: taehwa123@korea.kr

# Supplementary Tables

**Supplementary Table 1. Sweet potato genotypes used in this study**

| **Name** | **Country** | **Name** | **Country** | **Name** | **Country** | **Name** | **Country** |
| --- | --- | --- | --- | --- | --- | --- | --- |
| Bodueremi | KOR | Mokpo 50 | KOR | Chilbok | JPN | Ningshu 1 | CHN |
| Dahomi | KOR | Mokpo 56 | KOR | JA-1997-3 | JPN | Sang 52-7 | CHN |
| Geonhwangmi | KOR | Mokpo 58 | KOR | Jangbin | JPN | Wanshu 4 | CHN |
| Gogeonmi | KOR | Mokpo 75 | KOR | Kanto 48 | JPN | Xiangza 9 | CHN |
| Haenam jaerae 1 | KOR | Mokpo 77 | KOR | Kokei 14 | JPN | Xushu 18 | CHN |
| Happymi | KOR | Mokpo 83 | KOR | Minamiyutaka | JPN | Daenong 3 | TWN |
| Hogammi | KOR | Mokpo 87 | KOR | Nonglim 17 | JPN | Daenong 32 | TWN |
| Hopungmi | KOR | Mokpo 93 | KOR | Nonglim 22 | JPN | Daenong 54 | TWN |
| Hwangmi | KOR | Mokpo 108 | KOR | Nonglim 23 | JPN | Daenong 64 | TWN |
| Jangseong jaerae | KOR | Mokpo 117 | KOR | Nonglim 48 | JPN | Sacheonjong 36 | TWN |
| Jasaek sujip1 | KOR | Muan 1 | KOR | Norin 2 | JPN | SIS 5254 | TWN |
| Jeongeup jaerae | KOR | Muan 13 | KOR | Norin 7 | JPN | Suwon 109 | TWN |
| Jindo 4 | KOR | Muan 19 | KOR | Okinawa 100 | JPN | Suwon 110 | TWN |
| Jinyulmi | KOR | Naju jaerae 1 | KOR | Sazumahikari | JPN | V 21 | TWN |
| Juhwangmi | KOR | Pungwonmi | KOR | Su 2000 | JPN | V 63 | TWN |
| Mokpo 1 | KOR | Shinjami | KOR | Tsurunashi-genji | JPN | V 101 | TWN |
| Mokpo 2 | KOR | Sinhwangmi | KOR | Wongi | JPN | VISCA 37 | TWN |
| Mokpo 3 | KOR | Sugye 9 | KOR | Yen 158 | JPN | VISCA 83 | TWN |
| Mokpo 17 | KOR | Sugye 14 | KOR | Beijing 553 | CHN | VISCA 131 | TWN |
| Mokpo 19 | KOR | Sugye 16 | KOR | Chuan 8810-788 | CHN | VISCA 142 | TWN |
| Mokpo 22 | KOR | Yeonjami | KOR | China 10 | CHN | 70 iljo | TWN |
| Mokpo 36 | KOR | Beniharuka | JPN | Nanjing | CHN | AB95007-2 | IDN |
| Mokpo 37 | KOR | Benihayato | JPN | Nanjing 40 | CHN | Newzealandbam | NZL |
| Mokpo 44 | KOR | Benikomachi | JPN | Ning 210-18 | CHN | TIS 70357 | ETH |

**Supplementary Table 2. List of primers used in expression analysis of candidate genes**

| **Gene ID** | **Primer type** | **Primer Sequence (5'→3')** | **Amplicon size (bp)** |
| --- | --- | --- | --- |
| g12492 | Forward | GTGCAACACCCGAATTCTAC | 104 |
|  | Reverse | CGCACAACTTAGCAAGACCA |  |
| g12493 | Forward | GCTCCCGTGGTAGAAGAAGA | 125 |
|  | Reverse | CCTAGACCTGCCATTCCTTTG |  |
| g12494 | Forward | GGCTGTGAAGAAGGGTTTACTG | 105 |
|  | Reverse | TGAACAGTTAGGAGGGAGCTTG |  |
| g12495 | Forward | TGGTACATGATGGGTCGAAG | 142 |
|  | Reverse | GCTGATTCCTGAAAGCTACCC |  |
| g12497 | Forward | GTTTCAGTGAGGAGGATGACC | 80 |
|  | Reverse | GTAGGCCTCATTTCCTTGTCC |  |
| g13128 | Forward | TTGCTTCCGGGCTTTCTTTC | 81 |
|  | Reverse | CAAATCCCAACCAGAACCGG |  |
| g13129 | Forward | AGCAGGCCATGGAATCTGAA | 91 |
|  | Reverse | TGCATCCTATCGGCTCCTTG |  |
| g13132 | Forward | GAAATTCGCGGCGGAGATTC | 187 |
|  | Reverse | CTGGCACCTCATCTCCTTGA |  |
| g13133 | Forward | CTATGACAGGGCGGCATTTG | 85 |
|  | Reverse | TGATGCAGACGAAGGATGGT |  |
| g13134 | Forward | GGGAACATTCAACTCGGCTG | 92 |
|  | Reverse | CGGGGAAATTAAGGATGGCG |  |
| g13136 | Forward | CGGGACTTTGGTTTCACTGG | 138 |
|  | Reverse | ATCGAGCCTTTCACCCAGTT |  |
| g13143 | Forward | ACTGGCCACTCTGATCGTTG | 159 |
|  | Reverse | AGGGTTCGGCTCGCAATTAA |  |

**Supplementary Table 3. Descriptive statistics for Fusarium root rot resistance in the 96 genotypes**

| Year | Range | Mean | Standard deviation | Coefficient of variance (%) |
| --- | --- | --- | --- | --- |
| 2021 | 0.80 – 14.06 | 6.53 | 2.32 | 35.5 |
| 2022 | 0.05 – 14.27 | 6.71 | 2.62 | 39.0 |
| Combined years | 1.61 – 11.70 | 6.62 | 2.01 | 30.5 |

**Supplementary Table 4. Summary of mapped reads for the reference genome 'Taizhong6'.**

|  | **Raw reads** | | **Trimmed reads** | | | **Mapped reads** | |
| --- | --- | --- | --- | --- | --- | --- | --- |
|  | **No. of reads** | **Total length of reads (bp)** | | **No. of reads** | **Total length of reads (bp)** | **No. of reads** | **Mapping rate (%)** |
| Total | 662,568,562 | 100,047,852,862 | 519,705,096 | | 73,747,334,088 | 471,133,689 |  |
| Avg. | 6,901,756 | 1,042,165,134 | 5,413,595 | | 768,201,397 | 4,907,643 | 89.8 |
